# Risk factors for childhood obesity at age 5: *Analysis of the Millennium Cohort Study*

**DOI:** 10.1186/1471-2458-9-467

**Published:** 2009-12-16

**Authors:** Sinead Brophy, Roxanne Cooksey, Michael B Gravenor, Rupal Mistry, Non Thomas, Ronan A Lyons, Rhys Williams

**Affiliations:** 1 Centre for Health Information, Research and Evaluation, School of Medicine, Swansea University, Singleton Park, Swansea, SA2 8PP, Wales, UK; 2 Africa Educational Trust, 18 Hand Court, WC1V 6JF, UK; 3 Centre for Child Health, School of Human Sciences, Swansea University, Singleton Park, Swansea, Wales, SA2 8PP, UK

## Abstract

**Background:**

Weight at age 5 is a predictor for future health of the individual. This study examines risk factors for childhood obesity with a focus on ethnicity.

**Methods:**

Data from the Millennium Cohort study were used. 17,561 singleton children of White/European (n = 15,062), Asian (n = 1,845) or African (n = 654) background were selected. Logistic regression and likelihood ratio tests were used to examine factors associated with obesity at age 5. All participants were interviewed in their own homes. The main exposures examined included; Birth weight, sedentary lifestyle, family health behaviours, ethnicity, education and income.

**Results:**

Children with a sedentary lifestyle, large at birth, with high risk family health behaviours (overweight mothers, smoking near the child, missing breakfast) and from a family with low income or low educational attainment, were more likely to be obese regardless of ethnicity. Feeding solid food before 3 months was associated with obesity in higher income White/European families. Even when controlling for socioeconomic status, ethnic background is an important independent risk factor for childhood obesity [Odds ratio of obesity; was 1.7 (95%CI: 1.2-2.3) for Asian and 2.7 (95%CI: 1.9-3.9) for African children, compared to White/European]. The final adjusted model suggests that increasing income does not have a great impact on lowering obesity levels, but that higher academic qualifications are associated with lower obesity levels [Odds of obesity: 0.63 (95%CI: 0.52-0.77) if primary carer leaves school after age 16 compared at age 16].

**Conclusions:**

Education of the primary carer is an important modifiable factor which can be targeted to address rising obesity levels in children. Interventions should be family centred supporting and showing people how they can implement lifestyle changes in their family.

## Background

The World Health organisation (WHO) predicts that by 2015 approximately 2.3 billion adults will be overweight and more than 700 million will be obese. In 2005, at least 20 million children under the age of 5 were overweight [1]. Obesity can lead to many other health complications, including hypercholesterolemia and hypertension, and this can lead to serious health consequences. CVD and diabetes are two chronic diseases which are rapidly increasing globally. Even though the health consequences of obesity are most commonly seen during adulthood, the underlying factors of these diseases could originate during childhood. Weight at age 5 is a good predictor for further health of the individual and "the die seems to be largely cast by 5 years of age"[2]. Evidence is now emerging that obesity-driven type 2 diabetes might become the most common form of diabetes in adolescents within the next ten years [3]. It is therefore vital to know exactly how early the health consequences and risk factors for these serious diseases occur, and how early they can be detected if they are to be addressed successfully. This study investigates factors before age 5 to predict obesity at age 5.

The Millennium Cohort study (MCS) is a nationally representative cohort of British Children. Factors associated with being overweight were investigated when the children were age 3. Factors linked to higher weights were: higher birth-weight, black ethnicity, early introduction to solid foods, smoking during pregnancy, parental overweight. Protective factors included Indian ethnicity and longer duration of breast feeding [1]. Herein we examine risk factors occurring before age 5 to predict obesity at age 5 in children in the Millennium Cohort Study.

## Methods

### Millennium Cohort Study

This study used data collected by the Millennium Cohort Study (MCS)[4]. Data for this cohort were collected from children born over a 12 month period between 1 September 2000 and 11 January 2002. Families with children who were living in the UK at age 9 months and eligible to receive Child Benefit at that age were invited to participate (72% response rate)[5]. Subsequent interviews were carried out at the second contact (78% response rate) and third contacts (79.2% response rate) when children were approximately 3 and 5 years of age, respectively[5]. A stratified cluster sampling framework was employed to adequately represent families from disadvantaged areas and ethnic minority groups [5]. However, at second and third contact, there was a disproportionate loss of children from wards defined as 'ethnic' and 'disadvantaged'. Parents were interviewed in the home and over 99% of the main respondents interviewed were the biological mothers[6]. Obesity was defined using the International Obesity Task Force age and sex specific BMI cut offs [7]. Data from each of the three surveys were obtained from the UK Data Archive, University of Essex.

Sub-group of children examined: Only the singleton children (of the 18,552 children recruited at the first sweep of the MCS) were included within this study. The ethnic group of the child was recorded at the first contact using the 11 Category Census Classification (UK) and only children designated to either White, Asian or African ethnic group were included in the study (n = 17,561). The group defined as Asian included families recorded as; Indian, Pakistani, Bangladeshi and Other Asian, the group defined as African included families recorded as Black Caribbean, Black African and Other Black. Trained interviewers measured the children's weights and heights. Heights were measured using the Leicester stadiometer (Seca Ltd, Birmingham, UK) and recorded to nearest 0.1 cm and weight measurement was taken using Tanita HD-305 scales and recorded to nearest 0.1 kg. The primary outcome measure was obesity defined by the International Obesity Task Force (IOTF) cut-offs for BMI, which are age and sex specific [7].

### Potential Risk Factors

Factors associated with obesity were divided into categories and included risk factors found to affect obesity at age 3 using the MCS [1]. Risk factors examined include; eating habits (early introduction of solid food, irregular eating pattern, fruit consumption), activity, inactivity, family behaviours (mother's weight, smoking), birth weight, socio-economic status (income and education) and ethnicity. All these variables were collected from self-report by the respondent (usually the mother) and no food diaries or independent measurements of the parents (e.g. weight/height) were performed. Details of data collection are published elsewhere [5,6]. Breastfeed was not examined as this has been previously reported in detail using the MCS [8].

### Statistical analysis

STATA release 8 was used for all analysis. Factors associated with obesity were examined for evidence of confounding or interaction with income and length of time in education (as measures of socio-economic status) and ethnic background (White/European, South Asian or African) using Mantel-Haenszel tests followed by regression analysis using likelihood ratio tests. Logistic regression was performed using all factors associated with obesity in an initial unadjusted analysis and likelihood ratio tests were used to build the adjusted model. The interaction of all variables in the adjusted model with ethnicity was examined using likelihood ratio tests. Goodness of fit was assessed using the Hosmer and Lemeshow statistic [9]. Risk ratios for subgroups were also calculated to facilitate interpretation of findings.

## Results

In singleton births of White/European, Asian or African background the proportion of children in the obese category at age 5 was 5.7% (789/13745) and this rate differed significantly with ethnic background [see Table 1]. Lost to follow-up differed by ethnic group (Table 1) and by income; with 29%, 22% and 15% lost to follow up in the low (<£10,400), medium (>£10,400-£20,800) and high (£20,800+) income groups respectively.

**Table 1:** Risk fctors by ethnic group

	White/European (n = 15062)	Asian (n = 1845)	African (n = 654)
**Obese **% (95%CI)	5.4% (5.0%-5.8%)	7.1% (5.8%-8.6%)	11.7% (8.9%-15%)
		4.3% (2.6%-7% -Indian)	
		6.9% (5.2-9%-Pakastani),	
		11.7% (8.3%-16.2%- Bangladeshi),	
		5.4 (2.3%-12.1%-Other)	
Missing at age 5	3095 (20.5%)	514 (28%)	207 (32%)
			
**Income (at recruitment)**			
Less than £10,400	25% (24%-26%)	41% (38%-43%)	53% (48%-57%)
£10,400-£20,800	33% (32%-34%)	40% (38%-43%)	26% (23%-30%)
£20,800+	42% (41%-43%)	19% (17%-21%)	21% (24%-31%)
Missing	1050 (7.0%)	341 (18.5%)	108 (16.5%)
			
**Education (at recruitment)**			
No qualifications	16.5% (16%-17%)	45% (43%-48%)	33% (30%-37%)
O'level equivalent (education to age 16)	48% (47.5%-49%)	30% (28%-32%)	32% (28%-36%)
A'level equivalent (education to age 18)	10% (9%-10%)	8.6% (7%-10%)	8% (6%-10%)
University (education to age 20+)	25% (25%-26%)	16% (14%-18%)	27% (24%-31%)
Missing	251 (1.7%)	237 (12.9%)	50 (7.7%)
			
**Birth weight**			
Kg.(s.d.)	3.398 kg (0.57)	3.09 kg (0.59)	3.237 kg (0.63)
Missing	33 (0.3%)	8 (0.6%)	(1.2%)
			
**Solid food <3 months**	29% (28%-30%)	11% (10%-13%)	14% (12%-17%)
Missing	65 (0.43%)	35 (1.9%)	8 (1.2%)
			
**Enjoys physical activity (age 5)**	77% (76%-77%)	74% (72%-76%)	81% (77%-84%)
Missing	2983 (19.8%)	505 (27.4%)	198 (30.28%)
			
**Watches more than 3 hours of TV**	15% (14%-15%)	18% (16%-20%)	18% (15%-22%)
Missing	2980 (19.8%)	504 (27.3%)	200 (30%)
			
**Smoking near child**	16% (15%-16%)	6% (5%-8%)	5% (3%-7%)
Missing	2986 (19.8%)	507 (27.5%)	199 (30.4%)
			
**Mothers pre-pregnancy weight (kg)**	63.7 (12.5)	58.4 (11.5)	67.9 (14.1)
Missing	0	0	0
			
**For families where child is obese -Parent unconcerned about child's weight**	31% (28%-34.7%)	40% (29%-50%)	54% 41%-67%

### Eating habits

Unadjusted analysis showed that children categorised as obese at age 5 were more likely to have had first solid food before the age of 3 months, to have fewer portions of fruit per day, eat breakfast on fewer days of the week, to be less likely to eat at regular times compared to non-obese children [Additional File 1]. However, the effect of solid food before 3 months was most prominent in the higher income and white/European families [Additional File 1]. Eating breakfast was associated with lower levels of obesity in the families where the primary carer had a higher level of education [Additional File 1]. The association of obesity with fewer portions of fruit and with less regular eating times disappeared after adjusting for income.

### Physical activity

Crude analysis suggested that the children categorised as obese reported doing less exercise, and parents were less likely to report that the child enjoyed physical activity compared to non-obese children [Additional File 1]. However, the effect of reported exercise was no longer significant when adjusted for socio-economic factors. Surprisingly, although more than two thirds of respondents reported that their child enjoyed physical activity, there were low levels of actual physical activity reported (49% reported the child did no exercise per week - Additional File 1)

### Sedentary behaviour

Children who were obese at age 5 were more likely to watch more than 3 hours of television per day during the week compared to non-obese children and this association was consistent across different ethnic groups and at every level of income and education [Additional File 1].

### Indoor activities with child

Unadjusted analysis suggested that children categorised as obese at age 5 were more likely to do indoor activities such as reading, art and playing indoors with their parent than the non-obese children [Additional File 1].

### Family behaviours

Obesity at age 5 was found to be associated with a higher pre-pregnancy weight of the mother. This was consistent across all ethnic groups and across all levels of income and education. Obese children were more likely to be exposed to tobacco smoke compared to non-obese children but this association was modified by income, with no effect at low incomes but a risk ratio of 2.1 at higher incomes [Additional File 1]. One explanation for this could be that poor health behaviours with higher income could become more exaggerated with greater amounts of food consumed and more driving than walking to shops and schools. The association with smoking and obesity was consistent across ethnic groups.

### Socioeconomic factors

Obesity was associated with lower income levels and lower education levels of the main respondent. There was a non-significant trend among African families for higher prevalence of obesity with increasing income. However, there were too few subjects in each group to provide strong evidence of an effect [see Table 2]. In the high income African families, 16 out of 90 children (17% (95%CI: 11.2-27.0) were obese compared to 9% (95%CI: 4.8-16.2, n = 100) and 13% (95%CI: 9.1-19, n = 180) in lower income bands (£10,400-£20800 and <£10,400 respectively). In addition, the beneficial effects associated with higher educational levels in the mother, were less obvious in the African families [see Table 2].

**Table 2:** Obesity rates by ethnicity and risk factor.

	White/European n = 15062	Asian n = 1845	African n = 654
**Income**			
Less than £10,400	7% (6.1%-8.1)	8.7%(6.4%-11.8%)	13.3% (9.1%-19%)
£10,400-£20,800	5.5%(4.8%-6.3%)	6.2% (4.3%-8.8%)	9% (4.8%-16%)
£20,800+	4.5% (3.9%-5.1%)	5.4% (3.1%-9.3%)	17.8% (11%-27%)
			
**Education**			
No qualifications	7.1% (5.9%-8.4%)	9.2% (7%-12%)	11% (6.9%-17.5%)
O'level equivalent (education to age 16)	6.3% (5.7%-6.9%)	5.5% (3.5%-8.4%)	13% (8.9%-20%)
A'level equivalent (Education to age 18)	3.7% (2.8%-4.9%)	3.6% (1.4%-8.8%)	0% (denominator = 29)
University	3.5% (3.0%-4.2%)	5.5% (3.1%-9.6%)	
(Education to age 20+)			13% (8.7%-20.7%)

### Birth weight

Babies who were large at birth were more likely to be obese at age 5, This was consistent across ethnic groups and socio-economic levels [Additional File 1]. There was no association between obesity and low birth weight.

### Adjusted Regression analysis

Step up methods were used to enter all risk factors into one regression model. This model shows that ethnicity is an important independent risk factor even after accounting for income and education. Unadjusted odds of obesity in Asian children compared to White/European was 1.3 (95% 1.2-1.7) compared to the adjusted odds of 1.7 (95%CI: 1.2-2.3), unadjusted odds in African children was 2.3 (95% CI: 1.7-3.1) compared to the adjusted odds of 2.7 (95%CI: 1.9-3.9). Therefore, the odds of obesity in ethnic minority groups shows an increasing trend after controlling for the excess risks associated with low socio-economic status. The model suggests that there may be a U shaped curve with low income and high income families at higher risk of obesity then middle income families. The risks of obesity were highest among African and Bangladeshi children. However, children of Indian origin were not at higher risk of obesity at age 5 [Table 3].

**Table 3:** Regression analysis. Adjusted odds ratio

Risk factor	Odd ratio	95%CI
Ethnic group Asian (compared to White/European)	1.7*	1.2-2.3
Ethnic group African (compared to White/European)	2.7**	1.9-3.9
Birth weight (per kg increase in weight)	1.4	1.2-1.65
Does not enjoys physical activity (per unit increase (scale 1-5: 1 very much enjoys, 5 very much dislikes))	1.4	1.2-1.5
Watches more than 3 hours of TV a day	1.3	1.1-1.7
Solid food before 3 months	1.2	1.02-1.5
Smoking near child	1.3	1.02-1.6
Mothers pre-pregnancy weight (> 60 Kg)	1.9	1.6-2.3
Leaving school at age 18 (A'level) compared to leaving at age 16 (no qualifications or O'levels).	0.55	0.4-0.77
Leaving school at age 20+ (University) compared to leaving school at 16 (no qualifications or O'levels).	0.67	0.53-0.83
Income >£20,800 (compared to <£10,400)	0.84	0.67-1.0
Income £10,400-£20,800 (compared to <£10,400)	0.76	0.61-0.93
Indoor activities (per unit increase on a 0-30 scale).	1.06	1.03-1.08

* Risk Ratio = 1.6 (95%CI: 1.2-2.1). Indian = 1.1 (95%CI: 0.6-2.0), Pakistani = 1.5 (1.0-2.2), Bangladeshi = 2.1 (1.2-3.7), Other Asian = 1.6 (95%CI: 0.6-4.6).

** Risk Ratio = 2.5 (95%CI: 1.8-3.4)

Eating breakfast was no longer significant when all factors were entered. Interactions of ethnicity with: effect of solid food before 3 months; with enjoyment of physical activity; and with indoor activity and the interaction of income with smoking were no longer significant in a regression model when tested with log likelihood tests; odds ratios are given in Table 3. Goodness of fit assessed using the Hosmer and Lemeshow statistic [9] showed no significant difference between the model and observed data (p = 0.26), confirming a good fit of the model to the data.

## Discussion

### Main findings

Ethnic background is an important independent risk factor for childhood obesity. Many factors associated with obesity at age 5 are also similar across ethnic groups such as sedentary behaviour (hours watching TV), poor health behaviours in the family (smoking and pre-pregnancy obesity), low income and education, and large birth weight. Children from African or Asian families face not only higher risk but are also more likely to be exposed to the excess risk factors associated with lower socio-economic status. For example, African children have a risk ratio of obesity 2.5 times that of White/European children (odds ratio 2.7) controlling for socio-economics, and they are also twice as likely to live in a low income family where the mother has no qualifications. The trend to increasing odds ratios after adjusting for these confounding variables suggests that some aspects associated with low income could be protective for ethnic minority children. Some health behaviours which may be protective may be lost or changed with increasing income, for example walking to school declines with increasing income [10]. Higher educational level of the mother on the other hand is consistently associated with lower obesity levels. This maybe because with higher education there may be better uptake of protective health behaviours and empowerment for people to modify their own health. It is unlikely that education per se results in lower obesity but rather interventions which help people to implement healthy living advice and make modifications to their lifestyle may be the most effective for young families, for example, feeding style is associated with maternal education [11]. This is similar to findings looking at obesity at age 3 in MCS children, which support the conclusion that 'policies and interventions should focus on parents and providing them with the environment to support healthy behaviours for themselves and their children' [1].

In this study we found that feeding solids before 3 months is associated with higher BMI/obesity at age 5. This differs from findings looking at conditional weight gain between birth and age 3 using the MCS [8] which suggests that early introduction of solid foods has less effect on rapid gain once height is taken into account. However, the early introduction of solid food before 15 weeks was found to affect weight at age 7 [12] and is associated with subsequent unhealthy feeding behaviour [13]. Therefore, the effect of the early introduction of solid food on subsequent obesity is still debateable.

### Risk factors in ethnic minority groups

Ethnic minority children are at high risk from birth of obesity and many of these risk factors could be addressed. Differences between ethnic groups suggest beneficial effects of traditional methods such as not giving solid food before 3 months of age and enjoyment of physical activity. There is some evidence that obesity may not be seen as a problem and regarded as culturally acceptable among some groups. Specifically, 54% of African and 40% of Asian parents with an obese 5 year old reported to be unconcerned about their child's weight [Table 1]. There is a suggestion among the African groups that improvement in socio-economic status may bring about some increased risks of obesity and perhaps there is a U shaped curve with higher obesity levels in the poorer and wealthier families compared to middle income families. This finding is supported by the regression analysis which suggests that low and high income families (regardless of ethnicity) have similar risks of obesity and are both higher than the middle income families [14]. However, this hypothesis needs to be further examined. Some factors seen in ethnic minority groups are protective, and these practices may change with income. Similar to findings in this cohort when the children were age 3, Indian ethnicity was not found to be associated with higher levels of obesity [Table 3].

### Limitations of the study

The Millennium Cohort Study database provides a large sample size with high response rate and good representation of traditionally hard to reach groups. The Millennium Cohort Study is unique in over sampling ethnic minority groups and this is essential to ensure we have a fuller understanding of the ways of improving health and health behaviours across society. However, this study can only give a very crude assessment of the risk factors associated with obesity. For example, diet and physical activity are all self reported and open to variations in interpretation and meaning between different individuals and ethnic groups. No objective measures of diet (such as food diaries) or physical activity (such as accelerometers) were used in the study. The use of questionnaires to measure physical activity, especially parental reporting of physical activity is known to be problematic [15], overestimating the true levels of activity. Some of the factors associated with obesity given in this study, may be crude indicators of true risk factors. For example, missing breakfast in itself may not lead to obesity, but it may be an indictor of a general lifestyle of snacking, eating larger portions later in the day and a lack of thought on general diet [16]. The lack of objective measures for many of the explanatory factors examined means that residual confounding may remain. Recruitment to this study was through eligibility for Child Benefits. This method of identification could mean that migrant, refugee and asylum seekers are not included in this cohort and these may be the most vulnerable at risk groups for poor health care and obesity.

This study examines factors associated with obesity at age 5. However, there is the argument that thinness at age 5 may be more important for future health [17,18]. The rate of weight gain in the very early weeks of birth may be important for predicting future risk of obesity [19-21]. However, this study did not collect objective measures of weight gain between birth and 9 months of age.

Many of the findings within this study are supported by other cohort studies [22,23] which show babies large for gestational age, with mothers who smoked during pregnancy and who were large pre-pregnancy and low educational level, were more likely to be overweight. All these findings suggest that interventions targeting family and supporting people to implement lifestyle changes are promising to prevent childhood obesity.

## Conclusions

Obesity levels at age 5 vary significantly according to ethnic groups. Conversely, many risk factors and issues leading to childhood obesity are similar across ethnic groups. There is some evidence that increasing income may indicate some protective behaviours are lost. For example, earlier introduction of solid food and less physical activity are associated with higher incomes. However, higher educational achievement for the mother may be an important factor for addressing socio-economic inequalities in childhood obesity but this needs to be further explored for ethnic minority (especially African) families. Further work is needed to examine why some ethnic minority groups are at higher risk of childhood obesity. For example, variation between risks for Bangladeshi children compared to those from Indian families are not likely to be due to genetic differences. Interventions should be family centred maintaining the protective effects associated with a low income, but facilitating greater access to healthy choices associated with a higher income. This study uses the largest cohort of ethnic minority children and highlights that further work needs to be undertaken especially with African families who are at high risk of childhood obesity.

## Abbreviations

CVD: Cardiovascular Disease; MCS: Millennium Cohort Study; BMI: Body Mass Index.

## Competing interests

The authors declare that they have no competing interests.

## Authors' contributions

All authors were involved in designing the analysis, SB and RC obtained and analysed the data, MBG and RL advised on the analysis, RM, NT and RW give advice and comments on interpretation, all authors were involved in writing the manuscript and all authors have read and approved the final manuscript.

## Pre-publication history

The pre-publication history for this paper can be accessed here:

http://www.biomedcentral.com/1471-2458/9/467/prepub

## Supplementary Material

Additional file 1 **Evidence of association of risk factor with obesity**. Crude odds of association between exposure and obesity. Click here for file
