# Supplementary material for: Risk factors for childhood obesity at age 5: Analysis of the Millennium Cohort Study
Source: BMC Public Health. 2009 Dec 16;9:467. doi: 10.1186/1471-2458-9-467 (PMC2803190; doi:10.1186/1471-2458-9-467)
Supplement: Additional file 1 — Evidence of association of risk factor with obesity. Crude odds of association between exposure and obesity. [file 1471-2458-9-467-S1.DOC]

**Additional File 1.**

| **Exposure** | **% obese in exposed** | **% obese in unexposed** | **Odd ratio(exposed/unexposed)** | **Interactions** |
| --- | --- | --- | --- | --- |
| *Eating habits* |  |  |  |  |
| Solid food before 3 months | 250/3589 (7%) | 537/10,085 (5.3%) | 1.33 (95%CI: 1.13-1.55) | *Odds of obesity by ethnic group:* White/ European: 1.5 (95%CI: 1.3-1.8), Asian 0.45 (95%CI: 0.2-1.0), African 0.4 (95%CI: 0.2-1.1)  *Odds of obesity by income*: low income 0.9(95%CI: 0.7-1.2), Middle income 1.7(95%CI: 1.2-2.6), higher income 1.8(95%CI: 1.2-2.7) |
| 1 or less portions of fruit per day | 197/3013 (6.5%) | 589/10666 (5.5%) | 1.2 (95CI: 1.01-1.4) | - |
| Does not eat at regular times | 74/1025 (7.2%) | 712/12666 (5.6%) | 1.3 (95%CI: 1.02-1.7) | - |
| Eats breakfast less than 7 days per week | 95/1137 (8.3%) | 691/12546 (5.5%) | 1.6 (95%CI: 1.3-2.0) | *Odds of obesity by educational level:* no qualifications 0.93 (95%CI: 0.6-1.4), leaving school at 16 (O’level) 1.6 (95CI: 1.2-2.1), leaving school at 18 (A’level) 3.1 (95%CI: 1.3-7.8), leaving school at 20+ (University) 2.5 (95%CI: 1.4-4.5) |
| *Activity* |  |  |  |  |
| Enjoys physical activity | 738/13070 (5.6%) | 48/616 (7.8%) | 0.7 (95%CI: 0.5-0.95) | *Odds of obesity by ethnic group:* White/European 0.7 (95%CI: 0.5-0.96). Asian 0.8 (95%CI: 0.4-1.8), African too few numbers to estimate. |
| Exercising less than 1 day per week | 427/6696 (6.38%) | 359/6996 (5.1%) | 1.25 (95%CI: 1.08-1.45) | - |
| Watching more than 3 hours of TV per day | 159/2054 (7.7%) | 627/11633 (5.4%) | 1.47 (95%CI: 1.2-1.8) | - |
| Playing more than 3 hours of computers per day | 31/392 (7.9%) | 754/13296 (5.7%) | 1.42 (95%CI: 1.0-2.07) | - |
| *Indoor activities* |  |  |  |  |
| Parent plays indoors with child everyday | 223/3021 (7.3%) | 563/10,104 (5.6%) | 1.43 (1.2-1.7) | - |
| *Outdoor activities* |  |  |  |  |
| Playing physically active games outside with child everyday | 59/1258 (4.7%) | 447/7942 (5.33%) | 0.87 (95%CI: 0.66-1.15) | - |
| *Family Behaviours* |  |  |  |  |
| Mother pre-pregnancy weight >60kg | 604/8475 (7.13%) | 185/5270 (3.51%) | 2.1 (95%CI: 1.78-2.5) | - |
| Smoking near child | 147/1975 (7.4%) | 693/11710 (5.5%) | 1.39 (95% CI: 1.15-1.67) | *Odds of obesity by income:* Less than £10,400: 0.97 (95%CI: 0.71-1.3), £10,400-£20,800 : 1.47 (95%CI: 0.92-2.3) and £20,800+: 2.1 (95%CI 1.3-3.4) |
| *Socioeconomics* |  |  |  |  |
| Family income | Low – 237/3117 (7.6%)  Middle – 236/4190 (5.6%) | High – 253/5340 (4.74%) | 0.77 (95%CI: 0.7-0.85) | - |
| Leaving school at age 16 | 568/8432 (6.74%) | 192/ 4941 (3.89%) | 0.56 (95%CI: 0.47-0.66) | - |
| McClements below 60%median poverty indicator | 201/3152 (6.4%) | 379/7418 (5.1%) | 1.26 (95%CI: 1.06-1.5) | - |
| **Birth weight** |  |  |  |  |
| More than 3.5kg | 404/5792 (6.98%) | 385/7952 (4.84%) | 1.47 (95%CI:1.27-1.7) | - |
